# A Deep Learning Method for Fully Automatic Stomatal Morphometry and Maximal Conductance Estimation

**DOI:** 10.3389/fpls.2021.780180

**Published:** 2021-12-02

**Authors:** Jonathon A. Gibbs, Lorna Mcausland, Carlos A. Robles-Zazueta, Erik H. Murchie, Alexandra J. Burgess

**Affiliations:** ^1^School of Computer Science, University of Nottingham, Nottingham, United Kingdom; ^2^School of Biosciences, University of Nottingham, Loughborough, United Kingdom

**Keywords:** deep learning, g_smax_ – maximum stomatal conductance, high-throughput phenotyping, semantic segmentation, stomata

## Abstract

Stomata are integral to plant performance, enabling the exchange of gases between the atmosphere and the plant. The anatomy of stomata influences conductance properties with the maximal conductance rate, *g*_smax_, calculated from density and size. However, current calculations of stomatal dimensions are performed manually, which are time-consuming and error prone. Here, we show how automated morphometry from leaf impressions can predict a functional property: the anatomical g_smax_. A deep learning network was derived to preserve stomatal morphometry *via* semantic segmentation. This forms part of an automated pipeline to measure stomata traits for the estimation of anatomical g_smax_. The proposed pipeline achieves accuracy of 100% for the distinction (wheat vs. poplar) and detection of stomata in both datasets. The automated deep learning-based method gave estimates for g_smax_ within 3.8 and 1.9% of those values manually calculated from an expert for a wheat and poplar dataset, respectively. Semantic segmentation provides a rapid and repeatable method for the estimation of anatomical g_smax_ from microscopic images of leaf impressions. This advanced method provides a step toward reducing the bottleneck associated with plant phenotyping approaches and will provide a rapid method to assess gas fluxes in plants based on stomata morphometry.

## Introduction

Stomata are pores on a leaf that allow the exchange of gases between the atmosphere and the plant through their opening and closure (i.e., stomatal conductance – g_s_). Carbon dioxide (CO_2_) enters the plant in a trade-off against water vapour, which is simultaneously lost through transpiration. Stomata are found on almost all aerial plant organs and can be arranged in rows aligned with veins such as in monocotyledonous grasses or dispersed/clustered in dicotylodonous plants (Rudall et al., 2013). Their function is mediated by a pair of specialised cells, the guard cells, that control the aperture of the pore and determines the potential g_s_. As such, stomata are key “gatekeepers” positioned between the atmosphere and the internal plant tissue and are key in influencing photosynthetic rate, water loss, and water use efficiency (WUE) (Buckley, 2005; Berry et al., 2010). Stomatal morphology is diverse, with patterning (such as clustering), size and density reflecting inter- and intra-specific differences (Franks and Farquhar, 2007; Dow et al., 2014; McAusland et al., 2016), growing conditions (Casson and Gray, 2007), and evolutionary selection pressures (Franks and Beerling, 2009; Mcelwain et al., 2016). These anatomical characteristics have been shown to translate into functional diversity with, for example, size and density partly determining the leaf conductance capacity whilst the rapidity of guard cell movement determines the speed of response, or sensitivity, to environmental factors such as fluctuating light and water availability (Franks et al., 2015; McAusland et al., 2016; Bertolino et al., 2019). Indirect agronomic selection has been shown to lead to altered stomatal conductance in wheat (Fischer et al., 1998).

A measurement of stomatal size allows a calculation of the potential maximal rate of g_s_ to water vapour, known as anatomical maximum stomatal conductance (*g*_smax_; previously termed g_max_ or g_wmax_; Equation 1).


$$g_{smax}=\left(d.D.a_{\max}\right)/\left(v.\left(l+\left(\frac{\pi }{2}\right).\sqrt{\frac{a_{\max}}{\pi }}\right)\right)$$ Eq. 1

Where *d* is the diffusivity of water in air (m^2^s^-1^, at 25°C), *D* is stomatal density for a single leaf surface (mm^−2^), and *l* is pore depth (μm) and is estimated as half the mean guard cell width. For elliptical (i.e., graminaceous) guard cells, maximum stomatal pore area (*a_max_*; μm^2^) is estimated as an ellipsis with the major length estimated as pore length and minor length estimated as half the length of the peristomatal groove. For circular guard cells, *a_max_* is calculated as the area of a circle with diameter corresponding to the pore length. Finally, *v* is the molar volume of air (m^3^ mol^−1^ at 25°C), and *π* is the mathematical constant taken as 3.142 (Parlange and Waggoner, 1970; Weyers and Johansen, 1990; Franks and Beerling, 2009).

Anatomical *g*_smax_ often exceeds operational *g*_s_ by several fold (Sack and Buckley, 2016), but works in parallel with *g*_s_ at a spatial and temporal scale to optimise stomatal responses to the prevailing environmental conditions (Murray et al., 2020). High *g*_smax_ precludes high *g*_s_ under yield potential conditions and can be used to predict *g*_s_ under well-watered, light-saturated environments (Dow et al., 2014; Murray et al., 2020).

Improving the throughput and accuracy of measurements of stomatal size and density for the derivation of *g*_smax_ is essential, however, manual measurements of stomata are highly time consuming and small datasets are common when collecting images with few defects. Traditionally, stomatal density or index, the ratio of stomatal complexes to epidermal pavement cells, are collected through manual counting whereas measurements of pore and guard cell characteristics (morphometry) can be obtained through scaled dimensions using image processing software such as ImageJ (Schindelin et al., 2012). Whilst manual counts and morphometries are sufficient for smaller sample sets, they are untenable for screening larger populations – for example for genome-wide association studies (GWAS) – which often consist of 100s of lines with multiple replicates. Moreover, further issues arise in that they are susceptible to intra-rater or inter-rater repeatability (the subjective differences in measurements between individuals, or from a single individual repeating the same task), consequently reducing accuracy. One such solution to the limitations of manual morphometry can come from the field of neural networks, namely, deep learning. In deep learning, a computer model learns to perform classification tasks from images, text, or sound with a high degree of accuracy, sometimes exceeding human-level performance. The training of a deep learning model requires a human annotated dataset, which the model learns from and once trained, can be applied to future predictions, namely the same classification tasks on unseen data.

As of late, deep learning has received an increased amount of attention for both plant and stomatal phenotyping and various deep learning models have been proposed. With respect to stomata literature, the most common application of deep learning is for the detection and counting of stomata in images. Fetter et al. (2019) use a deep convolutional neural network (DCNN), AlexNet (Hinton et al., 2012), to generate a likelihood map for each input image followed by a thresholding and peak detection to localise and count stomata and achieved an accuracy of 94.2%. Zhu et al. (2021) use a Faster R-CNN combined with a U-Net to automatically count stomata and epidermal cells for the calculation of stomatal index and achieve 98.03 and 95.03% accuracy for stomata and epidermal cells, respectively. In other instances smaller, shallower, networks are used for counting; a convolutional neural network (CNN), VGG (named after the Visual Geometry Group where the method was conceived), is commonly used to detect each stoma, encapsulating the detections in bounding boxes (Simonyan and Zisserman, 2015). Meeus et al. (2020) use VGG19 in which the number (19) corresponds to the number of layers. Casado-García et al. (2020) use an object detection network known as YOLO (Redmon and Farhadi, 2018), to detect the bounding boxes of stomata with accuracy of 91%. Whilst good results are reported for detecting stomata using the VGG and YOLO networks, a considerable amount of post-processing is required if morphological measurements are to be extracted, which is susceptible to error. Alternatively, deep learning approaches have been used for the classification of stomata types; Andayani et al. (2020) created a CNN that determines whether the input image contains stomata from turmeric (also known as kunyit; *Curcuma longa*) or temulawak (also known as Java ginger; *Curcuma zanthorrhiza*). Using a small dataset of only ~300 images, they achieve classification accuracy of 93.1%. More recently DeepImageJ, a deep learning framework to plugin for ImageJ was released (Gómez-de-Mariscal et al., 2021). DeepImageJ provides significant advances of traditional methods and improves the capabilities of ImageJ, incorporating support for deep learning networks. Outputting high quality, accurate, classification of data, however, the specific results depend upon user design and implementation.

Current methods to comprehensively calculate stomatal morphometry are lacking and the limited studies to do so using a combination of deep learning and image processing. These methods typically focus on stomata detection *via* bounding boxes followed by image processing algorithms to obtain limited morphological data. However, these methods often require specific fine tuning where a change in intensity or blur within the image set will significantly reduce the accuracy. Toda et al. (2018) detect stomata, the pore and whether it is open or closed using a three-stage approach; (1) the use of the histogram of gradients (HOG) to detect stomata in the images and extract bounding boxes, (2) a CNN to classify the HOG detections as open or closed stomata, and (3) Pore quantification using a series of image processing algorithms, reporting accuracy of 92%. Bhugra et al. (2019) propose a framework consisting of two neural networks; the first, a DCNN, is used detect stomata in images, the second is a fully convolutional neural network (FCNN) which accepts the detected bounding box as input and extracts the stoma from the bounding box. Ellipse fitting is applied to the resulting FCNN output to generate an estimate of pore shape. Whilst producing good results, accuracy of ~91% for detection, ellipse fitting can over- or under-fit the pore. Moreover, instances where the pore is not ellipse shaped will lead to significantly inaccurate results. (c) use AlexNet to detect stomata and estimate pore area using a series of image processing algorithms [such as Contrast Limited Adaptive Histogram Equalisation (CLAHE)], achieving up to 85% accuracy. To date, both guard cell and pore measurements have yet to be obtained from a single network.

Semantic segmentation, in which each pixel of an image is labelled with a corresponding class, allows the preservation of morphometry. Unlike bounding box algorithms, the output in semantic segmentation is the image mask; a high-resolution image (typically of the same size as input image) in which each pixel is classified. Previous applications of semantic segmentation include, but are not limited to, medical imaging analysis (Jiang et al., 2018), autonomous driving (Siam et al., 2018), and classification of terrain from satellite imagery (Wurm et al., 2019). Despite the ability for semantic segmentation to extract morphometric information, it has yet to be applied to stomatal phenotyping.

Here, we aim to reduce the bottleneck associated with manually measuring morphometric traits of stomata and provide a proof of concept study for the determination of anatomical g_smax_ by the development of a high-throughput phenotyping method using semantic segmentation. We incorporate aspects of existing deep learning models, such as the Attention U-Net architecture (Oktay et al., 2018) and Inception Network (Szegedy et al., 2016), discussed in the *methods* section, on a small dataset (<350 images), whilst computational costs are reduced by restricting the number of the trainable parameters when compared to many of the existing deep learning methods for stomata. Through this method, we: (1) automatically differentiate between distinctive stomatal types, the dumbbell shaped Poacaea and dicotyledonous stomata, (2) count stomata, (3) extract multiple morphological traits, (4) calculate density, and (5) calculate anatomical g_smax_ as circular or ellipse based on the type of stomata. We provide a substantial advance with the application of semantic segmentation to stomata and the first to show deep learning can produce high-throughput stomata phenotyping calculating anatomical g_smax_. The tools developed here are freely available (See “*Data*” and “*Data availability*” sections).

## Materials and Methods

### Data

In highly researched areas, such as object detection or handwriting recognition, existing datasets such as ImageNet (Deng et al., 2010), or MNIST (Deng, 2012), provide access to hundreds of thousands of annotated images. In the case of stomata, however, very few annotated datasets are freely available.

Two balanced datasets with distinctive stomata were chosen to evaluate our proposed model: a monocotyledonous Poaceae representative with dumbbell shaped stomata (wheat; *Triticum aestivum*) and dicotyledon with kidney shaped stomata (poplar; *Populus balsamifera*). For the wheat set, spring bread wheat cultivars were chosen from the Photosynthesis Respiration Tails (PS Tails) Panel and from the International Maize and Wheat Improvement Centre (CIMMYT); with eight genotypes selected for their contrasting plant architecture and aboveground biomass that were grown under yield potential conditions in a glasshouse. A subset of the data was used in this study, consisting of 348 images captured at a resolution of 2,592×1,944px with a 10×40 magnification. The stomatal impressions were collected using nail varnish and adhesive tape in the medium area of adaxial and abaxial sides of the main shoot flag leaf. Samples were left to dry for 10min and then placed on a slide to be examined and photographed. Images were collected using a Leica DM 5000 B microscope (Wetzlar, Germany). The poplar dataset in this study was first published by Fetter et al. (2019) and is publicly available. A subset of the data was selected from an intraspecific collection of balsam poplar through random selection. A small subsample, totalling 114, images were annotated, which are of 2,048×2,048 px resolution with a 10×40 magnification. Note: the reduced poplar dataset used in this study, along with the corresponding annotations, has been made publicly available with links to the original source. The impression quality does not directly impact the quality of results unless the impressions used to train the network differ significantly from those used to test it. However, the quality should be good enough such that a human expert can manually annotate the images. Whilst the image set used for training was lower for the poplar, the increased density of stomata within each image led to a greater amount of stomata annotated overall (i.e., see Table 1).

**Table 1:** Overview of image datasets and properties.

Dataset	# Images	Size (px)	# Stomata	Density (mm2)	μm Per Pixel
Wheat	348	2,592 × 1,944	1,600	63	0.12547
Poplar	113	2,048 × 2,048	3,862	246	0.18181

**Figure 1:**
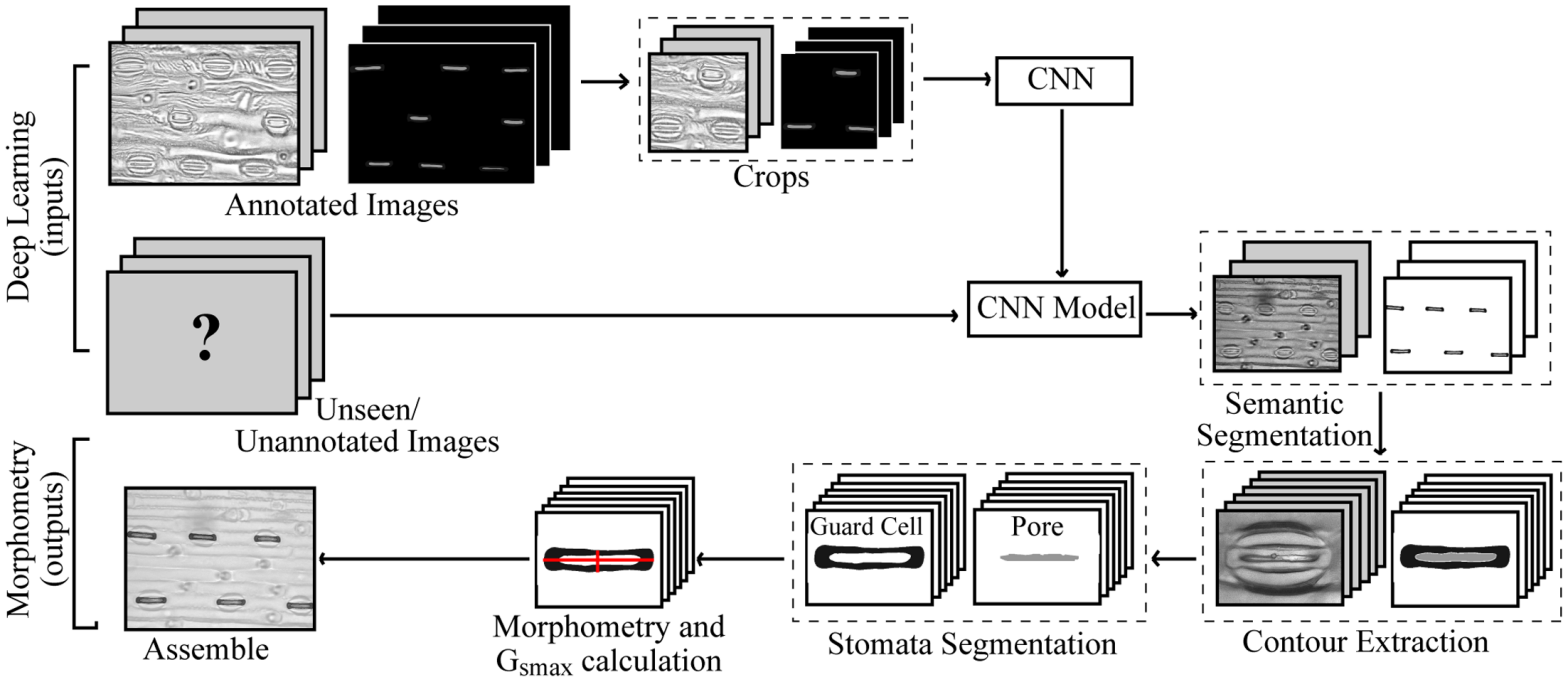
Pipeline for the proposed method of extracted morphometric properties of stomata for the estimation of maximal stomatal conductance; anatomical g_smax_.

An overview of the proposed method is given in Figure 1. For the annotation of both poplar and wheat datasets, a pixel level classification was performed where each pixel was labelled as guard cell, pore, or discard, to create the image mask using the Pixel Annotation Tool (Bréhéret, 2017). The discard refers to the background, noise (except that over the stoma), and subsidiary cells, which are not used in the calculation of g_smax_. Similar annotation approaches could be used for other structures, such as epidermal cells, trichomes or, on the whole plant scale, yield components for example.

### Data Augmentation

To increase the size and variation of the dataset, a series of augmentations were applied to manipulate the images prior to and during deep learning. Each original image is cropped into four overlapping regions as (1) the original image resolution is too large and is computationally expensive to maintain and (2) due to the small size of the pore within images, scaling the original image results in a high loss of accuracy. Augmentations within the network are applied for each image every epoch (a full iteration of the dataset) and are performed as follows; A subsample of the image is taken at a resolution of 768×768px. The centre of the bounding box, i.e., the area of the subsample, is determined by a series of random variables; the first randomly selects whether to perform a *stomata crop*; using the centre of the stomata, or a *random crop*; a random position within the image, with an 4:1 probability, respectively. The stomata crop randomly selects a stoma in the image and applies random jitter to the position with upper bounds of 15% of the image size. The random crop is selected anywhere within the image bounds excluding half the crop size around the border of the image. For both crop methods, a random rotation is applied ranging between plus and minus 30°. There is a 20% probability that the image will be flipped vertically or horizontally and a 30% probability of blur, sharpness, or contrast manipulation. These augmentations increase the dataset size and help prevent overfitting (where a network learns only the data it is being trained on). The augmentation is applied to the training dataset.

### Deep Learning

Within this project, all deep learning was performed using Python.

A brief overview of CNNs is provided for those who have no prior knowledge; for further reading, see (Maier et al., 2019). A CNN is a deep learning algorithm with a particular focus on imagery, for example, object detection or image classification within two-dimensional images. It is made up of a series of layers, each of which have a set of trainable parameters. The CNN takes as input an image and passes it through multiple layers and outputs a prediction that represents the class label of the input data, whether as an image as a whole or at pixel level. The three most common layers in a CNN are (1) *The convolution* layer which applies multiple filters, which aim to detect patterns such as edges, the input each of which have different parameters, so each filter is able to learn contrasting features whilst preserving the spatial relationship between pixels. The filters pass over the image, scanning a few pixels at a time, and creates a feature map. After a convolution, an *activation function* is performed to introduce nonlinearity calculating a weighted sum of its inputs and adding a bias. (2) The *Maxpooling* layer downsamples the feature map reducing its dimensionality, providing an abstracted form of the representation, and the associated computational costs. It allows for the CNN to be robust against minor displacements. (3) The final layer of a CNN is the *fully connected output* layer. After a sequence of multiple layers, it takes the outputs of these and classifies the pixels, computing scores for each class label applying an activation function such as *SoftMax*, which converts a set of numbers into a set of probabilities. Additional common steps can include *skip connections*, which allows the output of some layer to skip some other layers and be passed as input to layers further down the network.

The performance of a CNN, how well it has managed to learn these parameters and make predictions, can be evaluated in numerous ways. The *score* function or evaluation metric, evaluates the accuracy of the model during training, comparing the predicted outcome to the ground truth (i.e., the labels). The higher the score, the higher the degree of accuracy thus indicating that the model is correctly making predictions. The *loss* function is used as a method of evaluating how well the algorithm models the given data during training. If the predictions of a model deviate from the ground truth, a high loss value is returned. Too little data variation combined with a large network or high number of epochs can result in *overfitting*, where the model learns the training data and is unable to adapt to new or varying inputs.

**Figure 2:**
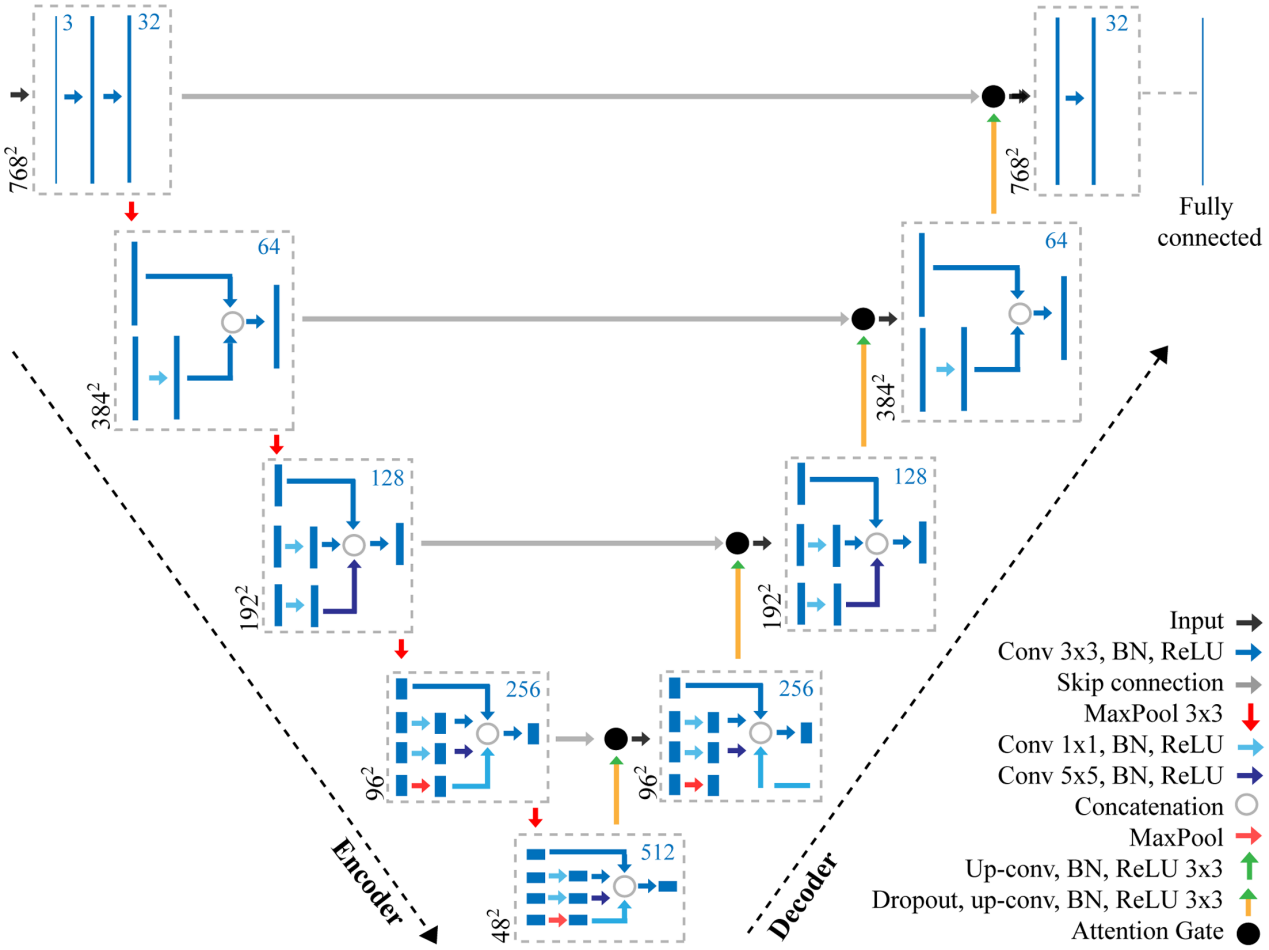
Overview of the adapted CNN used for the extraction of stomata morphometry. The proposed CNN combines features of both an Attention U-Net (Oktay et al., 2018) and Inception (Szegedy et al., 2016) to make pixel-level predictions of stomata for both guard cell and pore. The CNN contains a number of layers including convolution (Conv) layers, Max pooling layers, and fully connected layers. The output of each convolution layer is a set of 2D images, known as feature maps, which are computed by convolving previous feature maps with a filter, the size of which is given in the key. Batch normalisation (BN) and Rectified Linear Units (ReLU) steps are added to normalise data and remove negative pixel values from features maps. Skip connections help to maintain spatial information whilst the Attention Gate removes redundant features. The number of filters at each step is given as the blue number, whilst the resolution is given in black.

The structure of CNNs vary depending on the data, application, or the size of the network and so multiple networks exist. In this study, we propose a CNN using features of both an Attention U-Net (Oktay et al., 2018) and Inception (Szegedy et al., 2016) to make pixel-level predictions of stomata for both guard cell and pore (Figure 2). The original U-Net model (Ronneberger et al., 2015), which was primarily developed for biomedical image segmentation, is a U-shaped network comprised of a series of encoder and decoder layers. The encoder layer is the downwards trajectory performing a series of convolutions and maxpooling, encoding the input sequence (Figure 2). The decoder performs the opposite, an upwards trajectory applying deconvolution to increase dimensionality, decoding the input sequence to an output sequence. Skip connections are added between encoder and decoder layers to combine spatial information. However, whilst skip connections offer many advantages, such as the ability to maintain feature information, they introduce many redundant low-level feature extractions, as feature representation is poor in the initial layers. Attention U-Net overcomes this, expanding on the original U-Net model, by adding attention gates which seek to highlight salient features. Skip connections combined with attention gates suppress activations in irrelevant regions, reducing the number of redundant features. The inception architecture employs multiple convolutions and pooling layers simultaneously in parallel within the same layer (inception layer) using the same input. The inception layer reduces the computational costs of the model and automatically selects the most useful features when training the network.

Here, we present an incremental model (Figure 2), which increases the branches as the depth of the network increases, this works as follows.

- *Encoder layer 1*; The network takes, as input, an image with dimensions of 768×768 pixels. A Convolution (Conv) with a 3×3 filter, followed by Batch Normalisation (BN; normalises the input by re-scaling and re-centering the data, which increases the stability and speed of the network) and Rectified Linear Unit (ReLU; in which all negative pixel values in the feature map are converted to zero) is performed three times (we refer to this as Conv 3×3, BN, ReLU*x3*). Maxpooling is then applied with a kernel size of 3×3.
- *Encoder layer 2*; Receives input from the previous layer passing it through Conv, BN, ReLU*x2* followed by a maxpooling layer with a 3×3 kernel.
- *Encoder layer 3*; The input of the previous layer is copied into two branches, the first applies a Conv 3×3, BN, ReLU, whilst the second applies a Conv with a 1×1 filter, BN, ReLU followed by a Conv 3×3, BN, ReLU. The values are concatenated and a further Conv 3×3, BN, ReLU is applied. Maxpooling further reduces the dimensionality.
- *Encoder layer 4*; The input of the previous layer is passed to three branches, the first two are the same as the third encoder layer, whilst the additional branch performs Conv 1×1, BN, ReLU followed by a Conv 5×5, BN, ReLU. The values are concatenated and a further Conv 3×3, BN, ReLU is applied followed by maxpooling
- *Encoder layer 5*; Is the same as the previous encoder, but with an additional branch this time performing maxpooling with a 1×1 kernel followed by Conv 1×1, BN, ReLU.
- *Decoder layers 5–2*; Decoder layers 5–2 are the same as the encoder layers, though the maxpooling operation, which is used to down sample, is changed to a transpose convolution, which increases the dimensionality.
- *Decoder layer 1*; the final decoder, is responsible for the final output of the model and applies Conv 3×3, BN, ReLU*x3* followed by a fully connected layer to output predictions.

The parameters of the network were trained using Stochastic Gradient Descent (Kiefer and Wolfowitz, 1952) with a momentum of 0.9 and a learning rate of 0.1. The model was trained on an Nvidia Titan V GPU for 50 epochs using a batch size of 8. Whilst a GPU is not necessarily a requirement for deep learning, the speed of computations will be considerable using a CPU only. The Lovasz-Softmax (LS) loss function (Berman et al., 2018) is used; LS is a loss function for multi-class semantic segmentation incorporating SoftMax and supports direct optimisation of the mean intersection-over-union (IoU) loss in neural networks. IoU, also known as Jaccard index, is used to compute the area of overlap between the target mask (i.e., the annotated labels) and the predicted mask. The score function, or evaluation metric, evaluates the accuracy of the model during training. In this study, we use the IoU as a score function in two ways; (1) IoU is used to represent the percentage of overlap and (2) a confusion matrix summarises the performance of the model providing insight into the errors being made, returning an accuracy of the network. Moreover, the confusion matrix accounts for uneven number of samples for each class.

Once trained, the model allows new, unseen, images to be passed into the network producing, as output, a pixel-level annotation, the mask, of stomata within it. Unlike existing methods that use image processing methods to quantify the morphometry of stomata, in this study, the process is simplified by directly manipulating the mask. As a result, calculating morphometry becomes a relatively straightforward task, accomplished using a single network, and simple pixel counting.

### Stomata Morphometry

**Figure 3:**
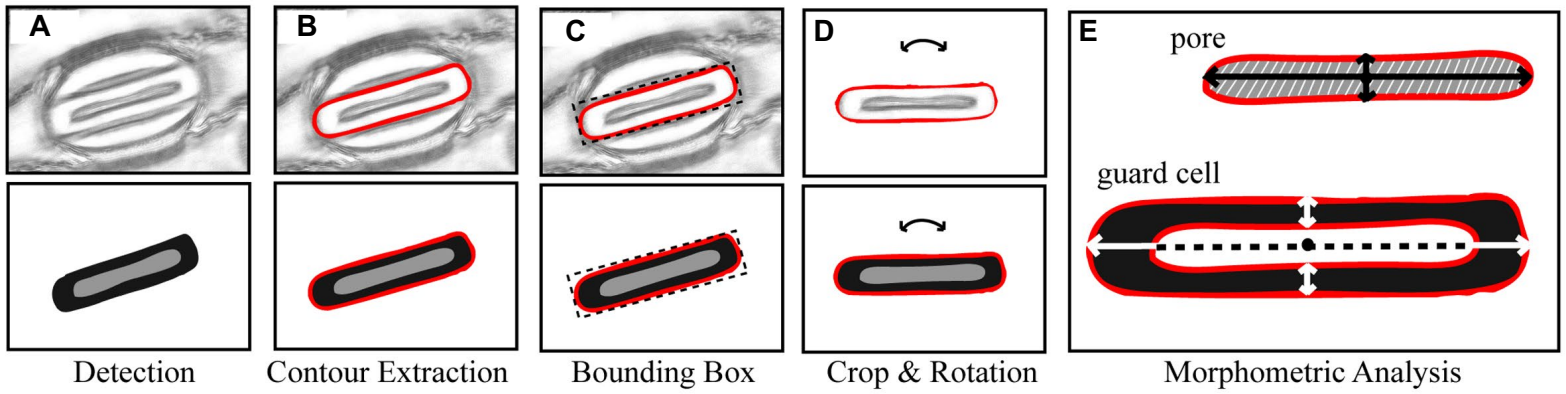
Overview of the stages of stomata morphometry extraction. **(A)** each stoma is detected using the CNN model described in Figure 1; **(B)** the contour is extracted; **(C)** a bounding box is applied to the contour; **(D)** the bounding box is rotated using the primary eigen vector and the stoma contained within the contour is cropped; and **(E)** morphometric measurements of the guard cell and pore are automatically extracted including guard cell and pore length and widths plus peristomatal groove distance.

Morphological traits such as length and width of pores can be segmented from the output of the CNN model proposed here by extracting information from the pixel-level labelled mask predicted by the CNN (Figure 3A). Contours in the mask are identified surrounding the guard cell (Figure 3B), and all pixels within each contour are selected and assigned to each individual stoma. A bounding box is fit around the contour and all background is removed (Figure 3C). Each individual stoma is rotated such that the principal axis is in line with the bounding box using the eigenvalues obtained from principal component analysis (Figure 3D). The rotation step supports the trait extraction; allowing widths and heights to be easily obtained. The mask is then split based on the corresponding label thus enabling the extraction of the pore from the stoma leaving the guard cell for automated morphometry (Figure 3E).

To calculate the morphometry of each stoma within the image, it is represented as a two-dimensional matrix where the values correspond to pore, guard cell, or discard. From this the width, height, and area of both the guard cell and pore can be calculated as a sum of pixels multiplied by the μm to pixel conversion. To obtain the measurements relating to the guard cell, the centre point, along both *x* and *y*, is selected and the length and width are calculated as the average sum of pixels along 10 pixel transects surrounding this centre point. This averaging is used to account for artifacts in the data (i.e., asymmetry in guard cell shape). The same process is applied to the pore.

Stomatal density is automatically calculated from the dataset. For all images, the number of stomata is counted, excluding any detected stomata, which intersects the left or bottom border of the image. The area within each image (i.e., the field of view; FOV) is calculated using a pixel to mm conversion (Equation 2)


$$FOV\left(mm^{2}\right)=\left(\frac{w*\mu m\;{\mathrm{pixel}}^{-1}}{1000}\right)*\left(\frac{h*\mu m\;{\mathrm{pixel}}^{-1}}{1000}\right)$$ Eq. 2

Where *w* and *h* correspond to the width and height of the image in pixels. Density, *D*, is then calculated according to Equation 3:


$$D=\frac{\mathrm{Total number of stomata}}{mm^{2}}$$ Eq. 3

Using these measurements, *g*_smax_ can be calculated using Equation 1.

## Results

The network was evaluated for its ability to accurately classify stomata type between wheat (Poaceae) and poplar in the datasets provided, detect features, obtain morphological traits, and predict g_smax_ compared to manual calculations.

### Stomata Detection

**Figure 4:**
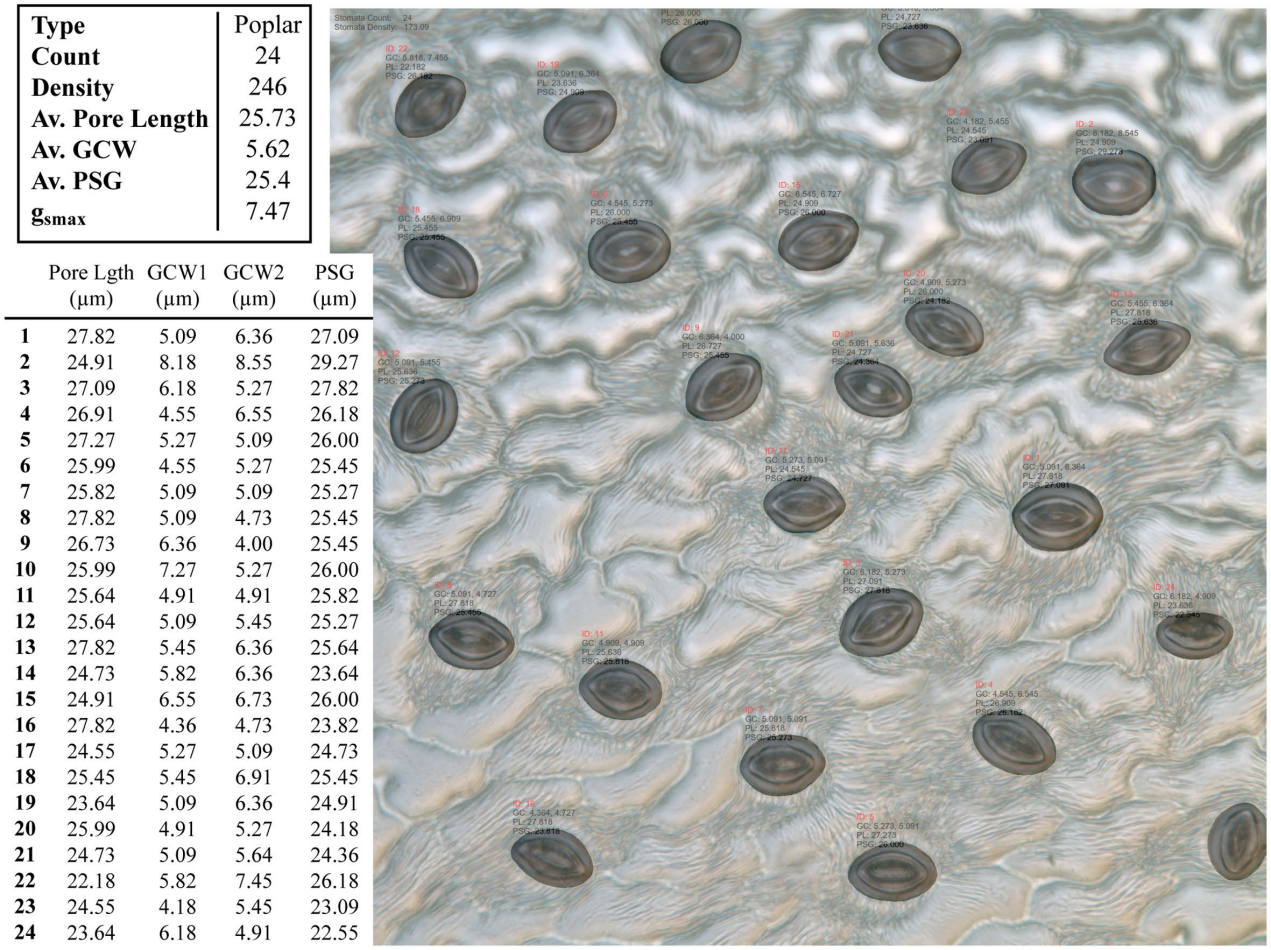
Example output from the CNN model applied to an unseen poplar image. Summary results for the whole images are given in the top table, whilst the measurements for individual stomata are given in the bottom, where GCW refers to guard cell width and PSG refers to peristomatal groove distance.

An example test image is presented in Figure 4 with the associated morphometric measurements.

The proposed network can be readily applied to both poplar and wheat, which have contrasting patterning (files vs. random spacing), thus making the method more universally applicable. The proposed model was evaluated against the U-Net (Ronneberger et al., 2015) and the Attention U-Net (Oktay et al., 2018) architectures. For each architecture, 25 epochs were performed using the same train and validation data. The results can be seen in Table 1; where *parameters* corresponds to the total number of trainable parameters in the network, *Time* is the total execution time in minutes, *IoU* is the intersection over union score; a value between 0.0 and 1.0 with 1.0 meaning that the prediction from the network is equivalent to the manual annotation, *Loss* is the result of the LS loss function, and *Acc*. is the accuracy of the model using a confusion matrix. As we can see from Table 2, the network proposed here has 50% fewer parameters than the related architectures, U-Net and Attention U-Net, and achieves at equal accuracy a higher IoU and a lower loss in a shorter amount of time.

**Table 2:** Comparison of the proposed convolutional neural network (CNN) relative to two other common CNN architectures.

Method	Parameters	Time (m)	IoU	Loss	Acc.
U-Net	~16,482,000	200	0.78	0.18	0.98
Attention U-Net	~17,450,000	343	0.72	0.18	0.97
Proposed method	~8,114,000	176	0.84	0.16	0.98

The number of parameters can have a direct impact on the computational cost of training a network and the future predictions made on unseen images. In most instances, a smaller number of parameters is preferable, particularly when access to high-spec hardware is limited. For that reason, we have reduced the parameters of the well-known U-Net architecture. The network proposed here has a total of ~8 million parameters, which is considerably less than existing approaches used for stomata deep learning, for example, the VGG16 network has ~138 million trainable parameters and the YOLO network has ~63 million. Here, we show that the number of parameters can be reduced whilst obtaining a higher degree of accuracy with our proposed method achieving 100% accuracy for stomata counts across both datasets. Moreover, no false positives, the prediction that a stoma is present when it is not, were recorded. If false positives were to be detected in images, the contour detection stage, discussed in the previous section, would discard any small errors based on average size of the stomata in the image.

### *g*_smax_

Manual calculations of morphometry for 20 images of both the wheat and poplar dataset were obtained by an expert, and the measurements were used to calculate g_smax_ using Equation 1. The images chosen were of various quality and spanning a range of examples from each dataset. These values were compared to those obtained using the automated method proposed here. One further benefit of the proposed CNN is that the stomatal type has been detected, and so g_smax_ can be calculated based on the most appropriate stomatal shape: circular for poplar or elliptical for graminaceous wheat stomata. It is worth noting that the difference here, between the predicted and manually determined measurements, is not classified as an error as the manual process is susceptible to intra-rater or inter-rater repeatability. To determine g_smax_ a series of variables need to be extracted from the data.

Stomatal density, given as an average across all images in the set, is given in Table 2, calculated using Equations 2, 3. In general, stomatal density is the biggest driver of variation in g_smax_, because the other two input variables (pore length and guard cell width) are averaged across many stomata and will differ less among samples. Within this proof of concept, the magnification required to calculate morphometry does not necessarily capture an accurate stomatal density as it will not cover a wide enough range of samples, thus the g_smax_ results presented here may differ from those reported elsewhere in the literature (Lammertsma et al., 2011). This is particularly the case for the poplar dataset whereby the obtained sample images were of fixed magnification and were originally collected to test a stomata counting system, focused on relatively stomatal-dense samples (i.e., leaf sections lacking vein structures etc.; Fetter et al., 2019). In contrast, the g_smax_ values calculated for wheat are likely more accurate because wheat stomata are patterned in rows and thus calculating density at 10×40 has less spatial bias. This can be overcome through the addition of more samples at this same magnification or through an additional step to count stomata at a lower magnification.

**Figure 5:**
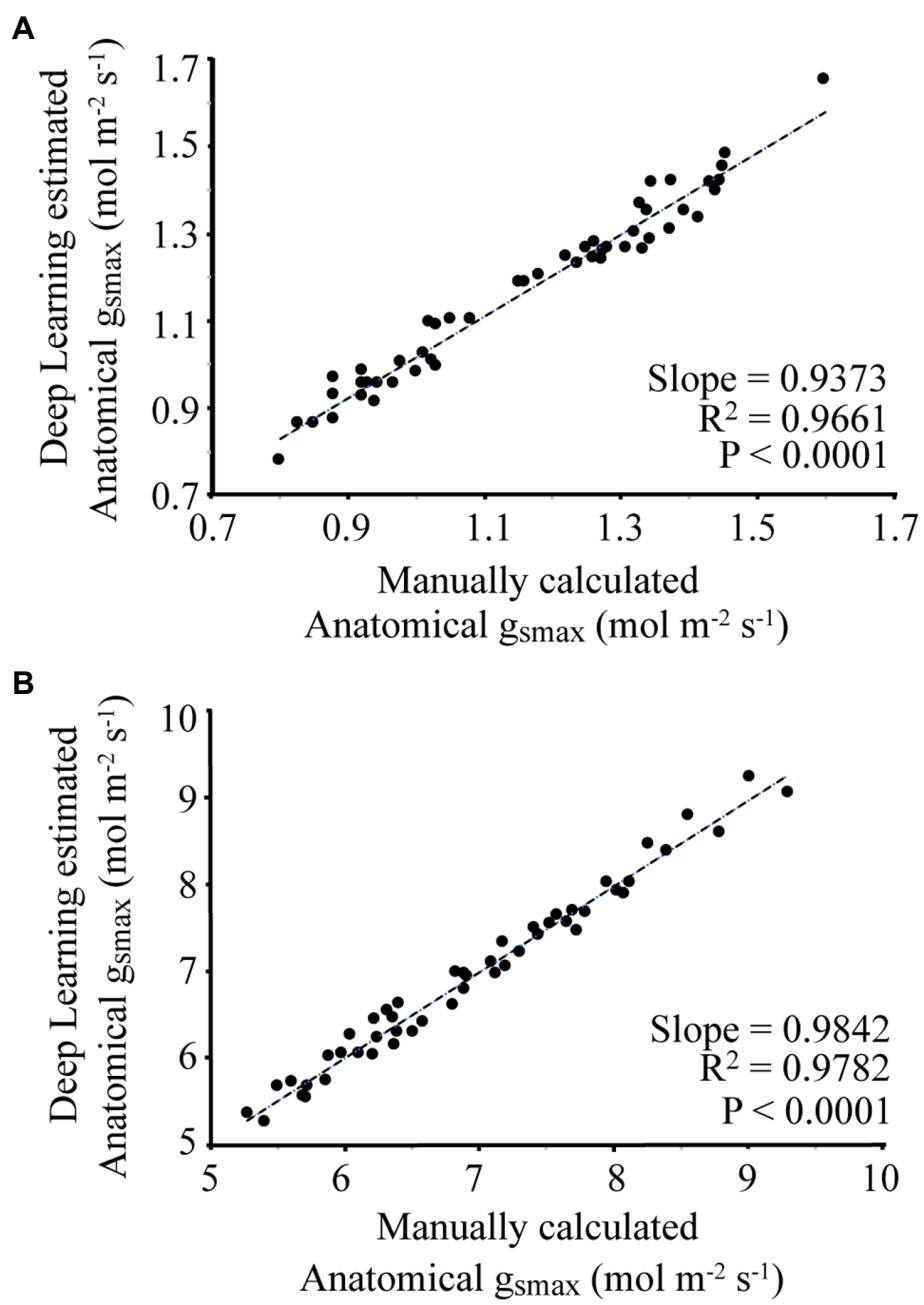
Comparison of manual calculation of g_smax_ by an expert vs. automatic calculation using the proposed deep learning approach where **(A)** corresponds to the wheat dataset, **(B)** is the poplar dataset.

For each image, the manually and automatically calculated g_smax_ is given in Figure 5. For the wheat dataset, the average difference between the manual and automated measurement was 3.8%, with a slope of 0.9373 and *R*^2^ of 0.9661. For the poplar dataset, the average difference between manual and automatic calculated g_smax_ was 1.9%, with a slope of 0.9842 and *R*^2^ of 0.9782.

This method also allows *operational g_s_* to be calculated on a per image basis, or over a set of images. Replacing $a_{\max}$ in Equation 1 with the area of the pore allows such calculations to be made.

## Discussion

Here, we present a significant advancement in methodology, which permits both morphological (density, size, and area) and functional (anatomical g_smax_) attributes to be predicted from purely image-based data that is easy to obtain and can be translated to high throughput systems. To our knowledge, this is the first time that the dumbbell – like Poaceae has been distinguished from dicotyledonous stomata and g_smax_ predicted using automated stomatal morphometry. Thus far g_smax_ has been used in disciplines where gas exchange measurements are inconvenient or simply not possible, for example, the recreation of conductance in palaeoclimates derived from fossils (Hetherington and Woodward, 2003; Franks and Beerling, 2009). This and similar approaches could prove useful in understanding and modelling future vegetation dynamics in climates with altered CO_2_ and water vapour. In the case of crop phenotyping, individual leaf gas exchange on large numbers of lines is impractical, making a functional prediction from image-based data invaluable. However, g_smax_ does not always correlate well with measured leaf g_s_ due to variation in aperture. Despite this, measurement of the actual pore area, as opposed to maximal pore area, permits the calculation of operational g_s_ (Dow et al., 2014). However, such comparisons require careful consideration of both the conditions of measurement and the accuracy of the pore area estimation from a two-dimensional image made using light microscopy. The stomatal guard cell complex should really be considered in three dimensions relative to the surrounding cell structure, with the possibility of sunken or raised pores, whilst thickening of the guard cell wall may blur the calculation of the actual pore area. Finally, the means of taking the impression itself leads to uncertainty: after the resin or varnish has been applied there is a period of many minutes needed for drying (depending on temperature), which has unknown effects on stomatal aperture. Thus, the calculation of operational g_smax_ requires care. If such problems can be overcome, then this method provides opportunities to predict function from purely morphometric analysis and may be amenable to in-field instrumentation. By linking operational g_smax_ with mechanistic models of leaf gas exchange and environmental conditions, a prediction of photosynthetic rate would become possible.

There is a vast amount of literature relating to the extraction of stomata data from 2D images, the most recent and relevant of which are presented and compared to the current method in Table 3. Accuracy is not directly compared as each individual approach uses a different dataset and methods vary between papers. Dependent on the phenotyping task, each of these methods could be of use however none of the approaches explicitly output a g_smax_ calculation, which relies upon pixel segmentation, orientation of the stomata, and individual measurements of pore and guard cell. Also the method presented here, whilst limited to stomata, offers a solution that requires no tuning of parameters or user interaction to determine the optimal network.

**Table 3:** Comparison of the proposed method and output compared to other recently published methods.

Method	Overview	Output
Proposed method	A convolutional neural network based on semantic segmentation and image processing tool for morphometric calculations of stomata plus the automatic estimation of g_smax_ Applied to Poplar and Wheat	Pixelwise detection Count Density Pore measurements Guard cell measurements g_smax_ estimate
Toda et al., 2018 DeepStomata	Developed software comprising histogram of gradients (HOG) detection of stomata followed by region classification by a CNN. Used for stomatal pore quantification. Applied to Dayflower	Pixelwise detection Count Density Classification between open and closed stomata Pore measurements
Bhugra et al., 2019	Detects and quantifies stomata using a CNN and a series of image processing techniques Applied to Rice using scanning electron microscopy (SEM) images	Bounding box detection Count Density
Fetter et al., 2019 StomataCounter	A CNN for counting stomata, which detects bounding boxes that encapsulate the stomata Applied to Ginkgo and Poplar	Bounding box detection Stomata count Density
Andayani et al., 2020	Uses a CNN and image processing for classifying stomata into one of two groups belonging to either turmeric or ginger	Classification
Casado-García et al., 2020 LabelStoma	Use YOLO (Redmon and Farhadi, 2018) to detect bounding boxes Applied to Common Bean, Barley, and Soybean	Bounding box detection Stomata count Density
Kwong et al., 2021	A CNN applied specifically towards detecting stomata from Oil Palm	Bounding box detection Count Density
Toda et al., 2021	A platform that supports real time stomata detection when directly connected to a microscope Applied to Wheat- *N.B. measurements of bounding boxes allow morphometric calculations of stomata when orientated parallel or perpendicular to the field of view*	Bounding box detection Count Density Bounding box measures
Zhu et al., 2021	Applies R-CNN, U-Net, and image processing to calculate stomatal index Applied to Wheat	Bounding box detection Counts of stomata and epidermal cells Stomatal index calculation
Gómez-de-Mariscal et al., 2021 DeepImageJ	A plugin for the widely used ImageJ application. Brings a sophisticated method for integrating deep learning with ImageJ. A user friendly interface which supports a wide range of phenotyping tasks	Dependent on the network but also on the user for defining and selecting the best choice for their needs. Will give detection and possible measurements but no automatic calculation of indices without an additional step

The proposed method provides many advantages over manually obtaining morphological measurements, not least the time in which it takes to calculate g_smax_. Unlike manual measurements, an automated approach allows for repeatability and a higher level of accuracy without bias, particularly beneficial for stomata phenotyping due to user-dependent variation in morphometric measurements. The time taken to calculate g_smax_ for a single image is less than a second regardless of the number of stomata present, substantially less than a manual approach. This may prove to be many hundreds of times faster with little manpower required. For example, it may take ~5–10min per sample to count manually, with longer timespans required to measure dimensions. In a high-throughput phenotyping context with many thousands of samples this is difficult or impossible to achieve with limited human resources. We improve on existing works achieving 100% accuracy for stomata counting and obtain *g*_smax_ results that are within 4% of the manual measurements calculated by an expert. Furthermore, the pipeline can be applied to different species or varieties, currently applicable to the poplar and wheat but easily expandable with addition of an increasing number of datasets.

Historical trends in stomatal density using herbarium specimens have shown that rising CO_2_ coincide with a reduction in stomatal density (Woodward, 1987; Hetherington and Woodward, 2003). Genetic manipulation has shown that changes in the size:density ratio can lead to changes in growth and WUE either through the improved uptake of CO_2_ or *via* reducing water loss (Lawson and Blatt, 2014; Franks et al., 2015; Bertolino et al., 2019). Recently, it was discovered that reducing frequency in multiple crop plant species resulted in an enhancement of WUE with no cost to photosynthesis or yield (Nadal and Flexas, 2019). Therefore, understanding and manipulating this relationship are vital for sustaining or improving crop yields under global climate change, especially in regions dominated by heat and drought conditions and where precipitation patterns are shifting, and advanced methods to automatically calculate this will become increasingly important (Prasad et al., 2008; Asseng et al., 2015; Hughes et al., 2017; Caine et al., 2019; Dunn et al., 2019; Mohammed et al., 2019). This will allow for the rapid identification of anatomical traits for multiple applications including the acceleration and exploitation of variation in large-scale crop populations, for example in heat and drought dominated regions where higher WUE is essential to increase crop yields, analysis of stored specimens such as herbariums and palaeobotanical samples (Araus et al., 2002).

### Application to General Research

The method presented here can be readily applied to new datasets. The key constraint, as with all deep learning methods, is the required annotated dataset; a network cannot find what it has not already “seen.” This can be easily accomplished using the Pixel Annotation Tool used within this study to manually classify the guard cells and pore (Bréhéret, 2017). The network itself was generated for novice users, although access to a graphical processing unit (GPU) is required. Sample files and further instructions can be found on github.^1^

Whilst it is still quicker and more efficient to annotate a dataset to apply to future samples, the obvious next step would be to reduce the bottleneck associated with manual annotations. Future work could look at the use of Generative adversarial networks (GANs; Goodfellow et al., 2014), which generate artificial annotations from a series of smaller datasets to reduce the overhead of training a network.

Previously stomatal conductance and related traits (i.e., transpiration, evapotranspiration, and photosynthesis) have been correlated in natural and crop ecosystems to remote sensing traits such as reflectance ratio R701/R820 as a response to photosynthesis and chlorophyll content in the leaves (Carter, 1998), Enhanced Vegetation Index (EVI), Normalized Difference Vegetation Index (NDVI), and Normalized Difference Infrared Index (NDII) in water scarce regions (Carter, 1998; Glenn et al., 2008; Joiner et al., 2018) or infrared thermography and water indices (Gutierrez et al., 2010). However, none of these remote sensing methods, whilst allowing direct means of assessing canopy function, permit a means of selecting specifically for stomatal anatomy traits, which must require analysis at the cellular level. The rapid estimations of g_smax_ proposed in this study can facilitate breeding programs especially in arid and semi-arid countries were WUE is the most important trait for yield improvement.

## Data Availability Statement

The datasets presented in this study can be found in online repositories. The names of the repository/repositories and accession number(s) can be found at: www.jonathongibbs.com/stomata2021 and www.github.com/drjonog.

## Author Contributions

JG, EM, and AB conceived the work. JG designed the model and computational framework, carried out implementation, performed the calculations and took the lead in writing the manuscript. CAR-Z collected the wheat dataset. JG and AB annotated the datasets. LM performed numerical calculations and assisted with stomatal biology. AB and JG designed and produced all figures for the paper. All authors contributed to the article and approved the submitted version.

## Funding

This work was supported by the Biotechnology and Biological Sciences Research Council (grant number BB/R004633/1). AB was supported by the Leverhulme Trust. CR-Z acknowledges financial support for his PhD scholarship to CONACYT, Mexico (CVU 626989). AB and JG wish to acknowledge support under the NVIDIA Academic Grant scheme.

## Conflict of Interest

The authors declare that the research was conducted in the absence of any commercial or financial relationships that could be construed as a potential conflict of interest.

## Publisher’s Note

All claims expressed in this article are solely those of the authors and do not necessarily represent those of their affiliated organizations, or those of the publisher, the editors and the reviewers. Any product that may be evaluated in this article, or claim that may be made by its manufacturer, is not guaranteed or endorsed by the publisher.

## Footnotes

^1^ http://github.com/drjonog
